# Course and Treatment of a Rare Neurological Sequelae After COVID-19: Miller Fisher Syndrome

**DOI:** 10.7759/cureus.29977

**Published:** 2022-10-06

**Authors:** Sam Kara, Michael A Wilson, Pamela Youssef, Kester Nedd

**Affiliations:** 1 Department of Neurology, Larkin Community Hospital Palm Springs Campus, Miami, USA

**Keywords:** covid-19, coronavirus disease, guillain-barré syndrome, intravenous immune globulin, miller-fisher syndrome

## Abstract

Reports of COVID-19 infection detailing its symptoms and outcomes point to its effects systemically, including that of the nervous system, such as the rare Miller Fisher syndrome (MFS). In this report, we identified a 43-year-old Caribbean man who arrived in the USA with ataxia and ascending bilateral lower extremity weakness after COVID-19 infection. Before arrival, the patient was diagnosed with Guillain-Barré syndrome (GBS). He was treated with IV methylprednisolone and a round of IV immunoglobulin (IVIG); however, he showed a minimal response. Upon admission to our ED, he had severe tachypnea and flaccid symmetrical quadriparesis combined with areflexia. Moreover, he had begun to exhibit signs of multiple cranial nerve palsies, including ophthalmoplegia and facial diplegia. Additionally, his laboratory cerebrospinal fluid (CSF) analysis was grossly normal. Therefore, he was diagnosed with MFS. Furthermore, he developed acute depression and exhibited signs of mania. The patient was treated with IV methylprednisolone and the second round of a five-day course of IVIG, resulting in marked clinical improvement. This case highlights the need for a multidisciplinary care approach in patients with MFS. It also points to the possible benefit of multiple IVIG rounds in MFS patients who do not improve after the first course.

## Introduction

The Guillain-Barré syndrome (GBS) variant, Miller Fisher syndrome (MFS), is a class of immune-mediated polyneuropathies which include the most common causes of acute or subacute acquired weakness. In some cases, it is complicated by respiratory failure and autonomic dysfunction. Thus, some patients may require hospitalization with intensive care [1,2]. Signs and symptoms of MFS, and GBS, usually begin in the lower extremities and spread progressively to involve the nerves of the upper extremities and torso. In addition, MFS involves the cranial nerves of the face and is characterized by the clinical triad of ataxia, areflexia, and ophthalmoplegia [3].

MFS is triggered as the humoral and cellular immune response cross-reacts with a shared antigenic determinant on peripheral nerves (molecular mimicry) [1]. The spectrum of these autoimmune syndromes is commonly provoked by an initial infection such as viral, bacterial, or post-vaccination [4]. Several post-infectious MFS and GBS cases are associated with COVID-19 [5]. In this case report, we present a patient with a COVID-19 infection post-vaccination, followed by MFS that improved following two rounds of IVIG.

## Case presentation

A 43-year-old male presented to our ED after arrival in the United States from Guyana with paraplegia and a positive COVID-19 polymerase chain reaction (PCR) test. He had a past medical history of hypertension, diabetes mellitus, and asthma. He had also been vaccinated against COVID-19 with the Sinopharm vaccine. Three weeks after his COVID-19 infection in Guyana, he rapidly developed severe generalized ascending weakness, dysphagia, and respiratory distress. Accordingly, the patient was diagnosed with GBS and was treated with three doses of methylprednisolone and a five-day course of IVIG. Unfortunately, the patient showed no improvement despite treatment and was transferred to our facility.

On admission, the patient had COVID-19 pulmonary symptoms, including shortness of breath, and was admitted to the ICU. His PCR test was also positive for COVID-19 infection. On initial examination, the patient complained of muscular ascending bilateral muscle weakness, lateral (0/5) more than proximal (1/5), dysphagia, fasciculations, and failed speech and swallow testing. He had bilateral absent deep tendon reflexes in the lower extremities with no sensory deficits. Furthermore, he started to develop bulbar symptoms, could not adduct the left eye medially, and had signs of facial spastic diparesis (ophthalmoplegia). The triad of ophthalmoplegia, ataxia and areflexia pointed to the MFS. He underwent cerebrospinal fluid (CSF) analysis, which revealed normal CSF protein (39.4 mg/dL), elevated glucose (82 mg/dL), and elevated polymorphonuclear and decreased mononuclear cells (Table 1). In addition, Ganglioside (GM1) antibodies, to check for a range of peripheral neuropathies, were negative. Finally, the patient underwent a non-contrast CT scan of the head to rule out stroke, which revealed normal brain anatomy with no changes.

**Table 1:** Cerebrospinal fluid analysis findings. CSF study was performed three times during the course of hospitalization to clarify the persistence of CSF findings during the course of illness. *CSF: Cerebrospinal fluid.

Test	Result	Reference
CSF total protein	39.4 mg/dL	18-45 mg/dL
CSF glucose	82 mg/dL	50-70 mg/dL
CSF cell count		
RBC	0.0 cells/µL	0.0 cells/µL
WBC	0.003 cells/µL	0.005 cells/µL
Polymorphonuclear (%)	33.3%	6.0%
Mononuclear (%)	66.7%	70.0-100.0%

The patient had been treated first with IV methylprednisolone 500 mg for one day and then a second five-day course of 0.4g/Kg IVIG. He also received Gabapentin 300 mg for neuropathic pain. On the sixth day of admission, he showed an excellent response by the end of the treatment. On day 12 of hospitalization, the patient started to improve clinically, and he was able to open his mouth, and his trapezius motor activity improved (4+/5). Furthermore, he was able to protect his airways with a full range of motion and strengthen his extensor cervical muscles. However, the patient's facial symptoms continued to worsen, and he developed an inability to close his eyes and bilateral facial weakness. In addition, the patient developed acute depression early on and throughout his ICU stay. Clinical signs of depression, without suicidal thoughts, were observed during the examination, and the patient was started on sertraline 50 mg for his depression. The patient passed his swallow evaluation test on day 17 and was discharged home with instructions to follow up with a neurologist, psychiatrist, and physical rehabilitation.

## Discussion

We present a rare post-COVID-19 neurological complication, MFS, in which the patient was diagnosed initially before being admitted to our service. The patient's clinical signs were consistent with MFS, even though the patient's CSF studies were within normal limits. Normal CSF studies are found in about 10% of patients with GBS and MFS [1]. This patient had level 2 MFS diagnostic certainty following the Brighton criteria, including the clinical triad of ataxia, areflexia, and ophthalmoplegia, the 11-day interval between illness and MFS, and CSF total white cell count <50 cells/μl [6]. The presence of the anti-gangliosides (GM1) has high sensitivity and specificity for MFS; however, this is not essential for the diagnosis [7]. Anti-GM1 was negative for this case, consistent with previous reports of MFS post-viral infection and with the majority of post-COVID-19 infections, suggesting there might be a missing antibody that has not yet been discovered [7,8].

It has been demonstrated that the SARS-CoV-2 virus binds to angiotensin-converting enzyme-2 (ACE-2) receptors prior to entry and infection of cells, and the virus may also enter a host cell via the RGD motif [9]. RGD, known as GD3, has cross-reactivity with GQ1b and is involved in the cell adhesion mechanism [10]. Besides MFS, anti-GQ1b immunoglobulins have already been linked to other etiologies, including Bickerstaff's brainstem encephalitis [1]. Anti-GQ1b immunoglobulins are part of the clinical manifestation of various pathogenesis of immunoglobulin-associated diseases, and the exact mechanism is yet to be determined. The anti-GQ1b immunoglobulins test was not available for our patient.

MFS patients are admitted to the ICU guided by the patient's clinical condition, including the severity of the symptoms and the respiratory muscle exhaustion manifesting as tachycardia, tachypnea, thoracic/abdominal muscle dyssynchrony, and the use of the accessory muscles for breathing [11]. These patients require mechanical ventilation, and 20-30% may require tracheostomy, based on at least one major criterion (such as PaCO2 >48 mm Hg, PaO2 <56 mm Hg, the vital capacity of <15 mL/Kg of total body weight, and negative inspiratory force < -30 cm H2O) or two minor criteria (such as poor cough reflex, dysphagia and chest X-ray showing lung atelectasis) [12].

The clinical presentation with bulbar palsy pointed to an MFS case, and our patient's neurological symptoms improved gradually following the IVIG second round. It is well known that those bulbar symptoms may take longer to resolve [13]. Our patient's bulbar signs were still present at the time of discharge, but he had successfully passed the speech and swallow test and was able to secure his airway. Moreover, MFS patients may develop chronic psychological distress as they sustain neuropathic pain and long-term disability, as evidenced in our patient [14]. Studies suggest that GBS patients have a 4.8-fold increased risk of developing major depressive disorder [15].

MFS treatment aims to lessen the severity of symptoms by pain control for neuropathic pain and ventilatory support if needed [16]. The two primary forms of treatment are immunoglobulin therapy and plasmapheresis. In addition, the same treatments used for GBS, such as IVIG, are indicated in severe MFS patients with swallowing difficulty and respiratory distress [1]. In addition, psychotherapy plays a pivotal role in treating MFS patients who develop psychological disorders and is one of the major concerns for patients and their families. Thus, this mandates professional and close monitoring by a multidisciplinary care team after discharge for a prolonged period [15].

In our case, the patient improved dramatically after receiving the second round of IVIG; however, currently, there is not enough data to support using the second round of IVIG in scientific guidelines. Still, new studies suggest efficacy, while others contradict this [17-19]. Therefore the role of multiple rounds of IVIG in post-COVID-19 MFS needs further investigation.

## Conclusions

There are few documented cases of MFS post-COVID-19 infection. In this case report, the classic clinical presentation and improvement support the diagnosis in the absence of the anti-GM1 autoantibodies, pointing to the involvement of a different autoantibody. Additionally, the patient's improvement following the second round of IVIG suggests that this could be a promising rescue therapy for patients with MFS post-COVID-19 infection, especially if they failed the first round of the IVIG regimen. However, more studies are needed to point to the missing autoantibody, if any, and support this treatment regimen. This case highlights the need for multidisciplinary care teams to manage patients with GBS and MFS.
